# Effects of Hatching Time on Behavior and Weight Development of Chickens

**DOI:** 10.1371/journal.pone.0103040

**Published:** 2014-07-24

**Authors:** Pia Løtvedt, Per Jensen

**Affiliations:** IFM Biology, AVIAN Behavioural Genomics and Physiology Group, Linköping University, Linköping, Sweden; CNRS (National Center for Scientific Research), France

## Abstract

The length of the embryonic period varies both among and within species and can affect the individual phenotype in many ways, both physiologically and behaviorally. In chickens, the hatch window may last 24–48 hours (up to 10% of the incubation time), and studies have shown that incubation length may affect post-hatch growth and physiology. However, little is known about effects on behavior. We therefore investigated how behavior variation correlates with hatching time in the early life of chickens. We also measured egg weight and egg weight loss in relation to hatching time, as well as post-hatch growth. For females, there was a negative correlation between hatch time and body weight from day 4 and throughout the experiment. For males, such a correlation was only observed when testing all hatched males up until day 10. The birds were exposed to a number of behavioral tests, and a principal components analysis was performed on the variables, resulting in four components. For the largest component, termed “Passivity”, a tendency of a difference was found between early and middle male hatchers. Furthermore, a significant difference between early and middle male hatchers was found in the second component, termed “Response to novelty”. In a spatial learning test, late hatchers tended to learn slower. The behavior of females was not significantly affected by hatching time in any of these tests. This study is among the first to demonstrate a link between time of hatching and early behavior in a precocial species like the chicken, and may help shedding light on the evolutionary trade-offs between incubation length and post-hatch traits. The results may also be relevant from a perspective of stress coping and therefore also for animal welfare and productivity in the chicken industry. The mechanisms linking hatching time with post-hatch phenotype remain to be investigated.

## Introduction

The length of embryonic development and the developmental maturity at birth vary extensively among different species. Altricial species have offspring which are born or hatched relatively immature and undeveloped and are highly dependent on parental care [1] (for example songbirds and parrots) while offspring of precocial species, such as chickens and ducks, are immediately able to move around and forage, and are not as dependent on their parents [1]. Between altricialness and precocialness, there is a continuum of varying maturity at birth, sometimes referred to as semiprecocial and semialtricial. Furthermore, developmental maturity at birth is correlated with gestation length, and in mammals, for a given maternal size, precocial species generally have a gestation length three times as long as altricial species [2]. In birds, although incubation is a costly behavior for the parent, a longer incubation period, leading to higher precociality, can be beneficial for the early survival capacity of the offspring. Thus, there may be a tradeoff between the benefits of the parent and offspring [3]–[5].

Differences in embryonic period length are not only seen between species, but also within species, in particular in ectoterms. For example, certain lizard species may show a variation in incubation length of more than 10 days for eggs in a single clutch [3]. Shine and Olsson [3] suggested that longer incubation time increases the maturity of lizards at hatch, and they were able to demonstrate higher locomotory speed in late hatchers. Furthermore, premature birth, as seen for example in humans, has been shown to give rise to both behavioral, cognitive [6] and physiological problems [7], as well as increased risks for a number of diseases [8]. Thus, it seems likely that also intraspecific variation may be crucial in shaping individual variation. In this experiment, we explore this for behavior and weight development in a precocial bird, the chicken (*Gallus gallus*).

Chicken eggs hatch after approximately 21 days of incubation, but within a single batch there may be a gap of 24-48 hours from the first to the last hatching, corresponding to 5–10% of embryonic development [9], [10]. This spread of hatch is often referred to as the hatch window [11]. Few studies have looked at length of incubation in a natural setting, and detailed knowledge of factors affecting hatching time of chickens is therefore mainly known from artificial incubation. It has been shown that intrinsic characteristics of the egg itself may have an effect, including age [12]–[14] and breed [12] of the mother, egg size [13]–[15] and sex of the chick [10], [16]. Interestingly, Hamilton and Hamilton [17] found that differences in timing of incubational stage arise even in the very first days of embryonic development. Furthermore, incubation practices may also influence the length of incubation. For example, length [16], [18] and temperature [12], [19] in storage before incubation, as well as incubator temperature [20], egg position [21] and turning conditions [22] have been found to be important. Hatching may also be stimulated by sound, such as clicking sounds from other eggs [23], and gaseous environment, more specifically, an increase in CO_2_ levels during incubation [24], [25].

Since prenatal environment, such as exposure to maternal hormones, has been demonstrated to affect the behavioral and physiological phenotype of the offspring in numerous ways [26]–[28], it is likely that the prenatal factors affecting incubation length, described above, may also affect the individual in other ways, for example altered timing of ontogenetic processes and ability to cope with stress [29]. Most studies investigating the effects of differences in hatching time of chickens have focused on post-hatch growth. Although many have found that there are no effects of hatching time on chick weight at hatch [10], , several studies have reported differences at later ages [10], [15], [18], [30], with late hatchers weighing less. Some studies also found physiological differences between early and late hatchers, such as different thyroid hormone levels and differences in organ weight and maturity [10].

Given the obvious importance of embryonic conditions on bird phenotypes, it is remarkable that there is very little knowledge concerning the effect of different hatching times on behavior. One study found that feeding behavior of broilers was affected by differences in hatch time, which partly explained post-hatch growth differences [30], but no studies have been performed investigating the effect of different hatching times on for example fear behaviors and learning, both crucial both from an evolutionary and an applied perspective. The aim of the present study was therefore to investigate the effects of early and late hatching on a number of behavioral traits in young chickens, as an example of a precocial bird species. We also measured egg weight, egg weight loss and post-hatch growth to assess any relationship between these variables and hatching time.

## Materials and Methods

### 2.1 Ethical note

The experiment and all its procedures were approved by the local ethical committee for animal experimentation in Linköping, approval no 122-10.

### 2.2 Animals: hatching and housing

Fertile eggs from the white commercial laying hybrid “Bovans” were purchased from a commercial breeder (Swedfarm, Linghem, Sweden). All eggs were collected on the day of purchase, so they were all laid by different mothers. They were transported to a hatching facility at Linköping University, and weighed, individually marked with a pencil and then incubated according to standards (37.5°C, RH 55%, with rotation).

At day 17 of incubation, the eggs were weighed again and moved to a hatcher with an IR sensitive video surveillance system and separation of eggs for individual hatching. Hatching occurred in total darkness, except for brief openings of the doors for chick collection (see below). The eggs were constantly filmed until hatching had taken place, and the videos were subsequently analyzed to determine time of hatching. The time of hatching was defined as time of emergence of the head from the egg.

During the hatching, the incubator was opened every 6 hours to retrieve hatched chicks. Hatched chicks were weighed and wing tagged, and then placed in a pen (0.75 m×1.50 m) with free access to food and water.

From hatching until the age of 13 days, all hatched chicks (N = 130) were kept together in the same pen. They were weighed on days 4, 7, 10 and 11.

At the age of 13 days, early, middle and late hatchers were selected for further tests. The 10 first, 10 last and 10 chicks closest around the mean hatching time for each sex were selected, making a total of 60 birds. These individuals were kept together in one group with 16 other birds from the same batch. The pen size was increased regularly as the chicks grew older, to a final size of 1.50 m×4.50 m at the age of 8 weeks. The remaining chicks were weighed again on day 14, and then every week throughout the rest of the experiment, until day 56.

### 2.3 Behavioral tests

From weeks 3 to 8, the chickens were exposed to a series of different tests to assess their fear behaviors and learning ability. All tests except for the tonic immobility test (see below) were monitored using a camera surveillance system, and behavior was automatically recorded using the software EthoVision (Noldus, version 3.1).

#### 2.3.1 Open Field (OF) test

At the age of three weeks, the chickens were exposed to an open field test (OF), designed to measure fear, anxiety and exploratory behavior [32]–[34]. The bird was placed in the corner of a dark novel arena measuring 80 cm×120 cm×120 cm. The arena was completely closed, so the birds could not see the observers or any parts of the experimental room outside the arena. The test was started by the lights being switched on, and the movements of the animal throughout the 5 minute test were recorded. For analysis purposes, the arena was virtually divided into center (40 cm×80 cm) and periphery. The variables recorded were latency to start walking, total distance moved, latency to enter center, and time spent in center.

#### 2.3.2 Social Reinstatement (SR) test

At the age of four weeks, the chicks were tested in a social reinstatement test (SR), measuring their social motivation [32], [35], [36]. They were placed in the corner of a dark runway arena measuring 40 cm×100 cm×120 cm. At the other end of the arena, two familiar stimulus animals, a male and a female randomly selected from birds in the same batch, which were not part of this experiment, were kept in a small compartment (40 cm×20 cm×30 cm) separated from the arena with wire mesh. A social zone (40 cm×30 cm) was virtually defined closest to the stimulus animals. The test was started by switching on the light. Each test lasted 5 minutes and the variables recorded were latency to start walking, total distance moved, latency to enter social zone and time spent in social zone. After every third test, the stimulus animals were changed to ensure that they stayed active.

#### 2.3.3 Novel Arena (NA) test, and Novel Arena Retest (reNA)

The novel arena test was a modification of the OF test, where the chicken was placed in a furnished arena and its exploratory behavior was recorded. It was performed two times for each individual to assess stability of the behavior, at the age of 5 and 6 weeks. To avoid habituation to the test arena, the floor of the arena was different between the two tests.

The birds were placed in the corner of a dark arena measuring 80 cm×120 cm×120 cm. Inside the arena, two boxes were placed (40 cm×20 cm×30 cm), 26 cm from the shorter wall and 20.5 cm from the longer wall. These boxes occluded parts of the arena from the birds view.

At the start of the test, the lights were switched on, and behavioral recording lasted 5 minutes. For analysis purposes, the arena was virtually divided into center (the area between the boxes), top zones (on top of the boxes) and periphery. The recorded variables were latency to start walking, latency to enter center, latency to enter top zones and total distance moved.

#### 2.3.4 Spatial Learning test

At the age of approximately 7 weeks, the spatial learning ability of the birds was tested. They were exposed to an arena with a simple maze five times during one day, and their time to reach the goal, a container with mealworms, was recorded in each trial. Each trial was separated by approximately 1.5 hours.

The maze was contained in a totally enclosed arena measuring 80 cm×120 cm×120 cm, and consisted of four lanes (80 cm×30 cm) separated by wire mesh walls with an opening (23 cm×30 cm) on alternating sides that the bird could pass through.

At the start of each trial, a bird was placed in the start corner of the arena, with the lights switched off. The trial started when the lights were switched on. Before the first trial, the birds were given mealworms to boost motivation.

In the first trial, the goal container with the mealworms was placed in the second lane, only separated from the birds by one wire mesh wall. The birds could see the mealworms, and only had to walk down the first lane and turn into the second lane to reach the goal. After successfully reaching the mealworms during the first trial, the goal was moved to the end of the fourth lane in subsequent trials. If the bird did not reach the goal during the first trial, the goal was not moved in the next trial. In each trial, the bird was allowed a total of 10 minutes to reach the goal.

The variables recorded in this test were latency to start walking and latency to reach the goal. In the data analysis, latency to start walking was subtracted from latency to reach goal to account for differences in individual fear levels.

#### 2.3.5 Tonic Immobility test

To test the fear level of the chickens, the tonic immobility test [34], [37], [38] was performed when the birds were 8 weeks old. The chickens were taken from their home pen and brought into a dimly lit room. They were placed on their back in a wooden cradle, and the person performing the test kept a soft pressure on the chest of the chicken for 5 seconds before removing the hand. If the chicken then stayed in the same position during 5 seconds, it was considered to be in tonic immobility. Otherwise, the induction attempt was repeated up to two more times. All tests were performed by the same person and in the same location. The number of induction attempts, the latency until the first head movement, and the latency until righting were recorded.

### 2.4 Data analyses

All data was tested for normal distribution using the Shapiro-Wilk test and for homogeneity of variance using Levene's test. Most variables were not normally distributed, and were therefore tested with non-parametric tests. Sex differences were assessed using Mann Whitney U test, whereas treatment differences were investigated with the Kruskal-Wallis (K-W) test. To be able to use exact hatching times of each individual to test for linear relationships with other variables, the Spearman rank test was used. For all statistical analyses, SPSS 20 was used. Weight variables and variables from the behavioral tests were subjected to two separate principal components analyses (PCAs) to reduce the number of variables used for statistical tests. Examination of the scree plots was used to determine the number of components to retain. The PCA scores for each individual were extracted, and all components were found to be normally distributed. Effects of hatching time were tested using two-way and one-way ANOVAs with Tukey's HSD post hoc test for pairwise comparisons. The components were also tested for linear correlations with hatching time using the Spearman rank test. Sex differences were tested using t-test.

In the spatial learning test, the effects of hatch group and sex on the number of animals that solved the task and the number of animals that reached the different were analyzed using a chi-square test.

For all tests, the criterion for statistical significance was set at p = 0.05. Values in the text are presented as means±standard error (S.E.) or as medians with interquartile range according to the type of statistical test used.

## Results

### 3.1 Hatching time

The first chick hatched after 487.8 hours of incubation, whereas the last hatched after 523.3 hours. Table 1 shows values for each hatch group for egg and hatch related variables. There was no difference in the mean hatch time of males and females (N = 130, U = 2251, p = 0.46), nor did time of hatching correlate with egg weight (r_s_ = 0.10, p = 0.24, N = 130), absolute egg weight loss (r_s_ = -0.03, p = 0.78, N = 130), percent egg weight loss (r_s_ = -0.10, p = 0.27, N = 130) or weight at hatch (r_s_ = 0.004, p = 0.96, N = 130). However, hatch weight relative to egg weight was negatively correlated with hatch time, so embryos with large relative weights hatched earlier (Relative to egg weight at onset of incubation: r_s_ = -0.29, p = 0.02; Relative to egg weight at embryonic day 17: r_s_ = -0.34, p = 0.01, N = 130).

**Table 1:** Means and standard errors of the measured egg and hatch variables for the three different hatch times (N = 60).

	Hatch time
Variable	Early	Middle	Late
Hatch time (h)	491.7±0.4^a^	498.8±0.1^b^	506.3±1.1^c^
Egg weight D0 (g)	60.2±0.7	59.3±0.8	61.0±0.8
Egg weight D17 (g)	54.9±0.6	54.3±0.7	55.9±0.8
Egg weight loss (%)	8.66±0.18	8.50±0.23	8.45±0.32
Egg weight loss (g)	5.2±0.1	5.1±0.2	5.1±0.2
Hatch weight D0 (g)	43.2±0.5	42.4±0.6	43.2±0.6

Within rows with superscripts, values sharing no common superscript were significantly different.

Within each sex, hatch time did not correlate with egg weight, absolute or relative egg weight loss or weight at hatch. In males only, there was still a negative correlation between hatch time and hatch weight relative to egg weight (Relative to egg weight at onset of incubation: r_s_ = -0.24, p = 0.05; Relative to egg weight at embryonic day 17: r_s_ = -0.30, p = 0.01, N = 71). This correlation was not seen in females only (Relative to egg weight at onset of incubation: r_s_ = -0.17, p = 0.20; Relative to egg weight at embryonic day 17: r_s_ = -0.15, p = 0.26, N = 59).

### 3.2 Body weight PCA and correlations with hatch time

**Figure 1:**
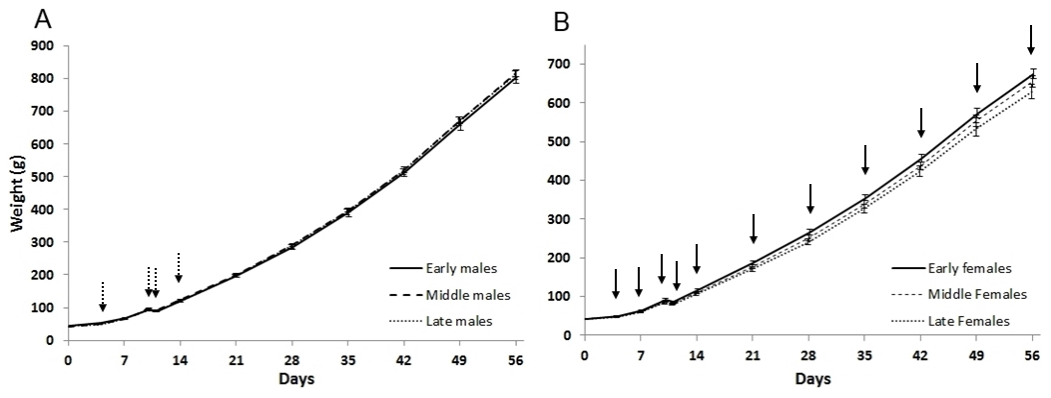
Weight development of a) males (N = 30) and b) females (N = 30) over the experiment. The graphs show mean±S.E for each hatch group. The solid line arrows indicate significant correlation (p<0.05) between hatch time and weight, whereas the dashed arrows indicate tendency (p<0.1).

Figure 1 shows the weight development of males and females in the three hatch groups. On day 4 after hatching, there was a negative correlation between the hatching time and the body weight of both males (r_s_ = -038, p = 0.001, N = 71) and females (r_s_ = -0.31, p = 0.02, N = 57). In females, this correlation was observed for all hatched females on days 4-10 (r_s_ = -0.28 to -0.34, all p<0.05, N = 56-58), as well as a tendency on day 11 (r_s_ = -0.23, p = 0.09, N = 58), and also for the 30 females that were chosen for subsequent behavioral tests (days 4-56, r_s_ = -0.37 to -0.51, all p<0.05, N = 29-30).

In males, the whole group of hatched males displayed such a correlation on days 4, 7 and 10 (r_s_ = -0.24 to -0.38, all p<0.05, N = 69-71), and a tendency on day 14 (r_s_ = -0.21, p = 0.08, N = 71). The males selected for behavioral tests only showed tendencies on days 4, 10, 11 and 14 (r_s_ = -0.33 to -0.34, all p<0.10, N = 29-30), but not on day 7 and not after day 14.

To reduce the number of weight variables, a PCA was performed. Two components were extracted, accounting for 89.2% of the variance in the dataset (Table 2). All weight variables apart from weight at hatching loaded heavily on the first component, whereas the second component included only variables from the first two weighings.

**Table 2:** Weight variable loading scores and variance explained for the two components extracted in the PCA for weight.

	Components	
Variable	1	2
Hatch weight D0		0.66
Weight D4	0.72	0.56
Weight D7	0.90	
Weight D10	0.91	
Weight D11	0.92	
Weight D14	0.95	
Weight D21	0.97	
Weight D28	0.96	
Weight D35	0.94	
Weight D42	0.93	
Weight D49	0.91	
Weight D56	0.88	
% of variance	76.5	12.6

Only loadings with an absolute value >0.4 are shown.

Individual scores were calculated, and analyzed for correlation with hatch time. For component 1, there was a negative correlation with hatching time (r_s_ = -0.29, p = 0.03, N = 58) when combining both sexes, whereas for component 2, there was no correlation (r_s_ = -0.19, p = 0.16, N = 58). Splitting the data in males and females revealed no correlation between hatching time and the weight components of males, whereas the females displayed a significant negative correlation between hatching time and component 1 (r_s_ = -0.48, p = 0.01, N = 29), but not between hatching time and component 2 (r_s_ = -0.20, p = 0.31, N = 29).

### 3.3 Behavioral variables and PCA

Table 3 shows values for the behavioral variables for each hatch group. When considering both sexes together, the only variables differing between the groups were SR Time spent in social zone (Medians: Early = 21.3 s, Middle = 0.0 s, Late = 8.6 s. K-W: χ2 = 6.2, p = 0.05), SR Latency to enter social zone (Medians: Early = 262 s, Middle = 300 s, Late = 280 s. K-W: χ2 = 6.6, p = 0.04) and NA Latency to enter center (Medians: Early = 300 s, Middle = 300 s, Late = 300 s. K-W: χ2 = 6.4, p = 0.04).

**Table 3:** Hatch group medians (interquartile range) for each of the behavioral variables measured.

	Hatch group
Variable	Early	Middle	Late
OF Time in center (s)	86.8 (56.4)	79.9 (71.5)	76.2 (59.1)
OF Latency to enter center (s)	97.2 (69.5)	79.4 (48.6)	110.3 (61.0)
OF Latency to start walking (s)	63.5 (64.5)	48.5 (36.8)	81.5 (53.3)
OF Total distance moved (cm)	501.6 (495.1)	653.2 (519.8)	680.2 (409.9)
SR Time spent in social zone (s)	21.3 (64.9)^a^	0.0 (0)^b^	8.6 (71.8)^a^
SR Latency to enter social zone (s)	262.0 (112.6)^a^	300.0 (0.0)^b^	280.4 (96.2)^a^
SR Total distance moved (cm)	104.8 (170.8)	22.0 (153.88)	83.6 (250.7)
SR Latency to start walking (s)	86.5 (83.3)	226.0 (210.0)	133.5 (208.8)
NA Latency to enter center (s)	300.0 (96.3)^a^	300.0 (0.0)^ab^	300.0 (0.0)^b^
NA Latency to enter top zone (s)	300.0 (120.9)	267.4 (165.1)	246.1 (200.9)
NA Total distance moved (cm)	317.8 (530.2)	312.5 (396.9)	339.4 (553.3)
NA Latency to start walking (s)	35.5 (77.8)	65.5 (97.3)	52.0 (92.0)
reNA Latency to enter top zone (s)	253.2 (185.0)	300.0 (0.0)	300.0 (190.3)
reNA Latency to enter center (s)	300.0 (0.0)	300.0 (0.0)	300.0 (0.0)
reNA Total distance moved (cm)	61.2 (110.7)	33.9 (147.2)	15.8 (155.3)
reNA Latency to start walking (s)	132.5 (135)	156.5 (243.0)	97.5 (251.0)
TI Latency to head movement (s)	20.5 (27.8)	34.0 (53.0)	33.5 (84.3)
TI Latency to righting (s)	33.5 (84.3)	76.0 (74.0)	53.0 (104.3)

Tests used were Open Field (OF), Social Reinstatement (SR), Novel Arena (NA), Novel Arena Retest (reNA) and Tonic Immobility (TI).

Within rows with superscripts, values sharing no common superscript were significantly different.

However, in males only, there were significant differences between the hatch groups for the variables SR Latency to enter goal zone (Medians: Early = 235 s, Middle = 300 s, Late = 268 s. K-W, χ2 = 6.5, p = 0.04), SR Latency to start walking (Medians: Early = 74 s, Middle = 246 s, Late = 191 s. K-W: χ2 = 6.9, p = 0.03), TI Latency to head movement (Medians: Early = 9 s, Middle = 65 s, Late = 39 s. K-W: χ2 = 7.6, p = 0.02) and reNA Latency to enter top zone (Medians: Early = 115 s, Middle = 300 s, Late = 300 s. K-W: χ2 = 6.8, p = 0.03), as well as a tendency of a difference in SR Time spent in social zone (Medians: Early = 48 s, Middle = 0 s, Late = 0.7 s. K-W: χ2 = 6.0, p = 0.05).

In females only, no significant differences or tendencies were found in any of the behavioral variables.

The novel arena test was performed twice to assess stability of behavior. Except for Latency to enter center, all other variables were significantly correlated across the two tests (r_s_  = 0.40 to 0.46, all p<0.003).

To reduce the number of behavioral variables for the statistical analysis, a PCA was conducted. Four components, explaining a total of 62.9% of the variance, were extracted (Table 4). The first component had high loadings from variables total distance moved and latency to start moving in different tests. The component was therefore called “Passivity”. The second component was termed “Response to novelty”, as variables loading heavy on this component were mainly from the OF test. The third component was termed “Exploration”, whereas the fourth was called “Anxiety”.

**Table 4:** Behavioral variable loading scores and % variance explained for the four components extracted in the behavioral PCA.

	Components			
Variables	1	2	3	4
OF Time in center		0.55		
OF Latency to enter center		−0.85		
OF Latency to start walking	0.43	−0.67		
OF Total distance moved	−0.44	0.43		
SR Time spent in social zone	−0.67			0.48
SR First entry in social zone	0.71			−0.52
SR Total distance moved	−0.67			
SR Latency to start moving	0.78			
NA Latency to enter center				0.45
NA Latency to enter top zone	0.52			
NA Total distance moved	−0.51	0.40		
NA Latency to start moving	0.76			
TI Latency to first head movement		0.49	−0.45	0.57
TI Latency to righting			−0.45	0.51
reNA Latency to enter top zone	0.48		0.45	
reNA Latency to enter center				
reNA Total distance moved	−0.56		−0.55	
reNA Latency to start moving	0.55		0.65	
% of variance	25.9	14.9	10.9	10.3

Only loadings with an absolute value >0.4 are shown. Abbreviations are explained in Table 3.

**Figure 2:**
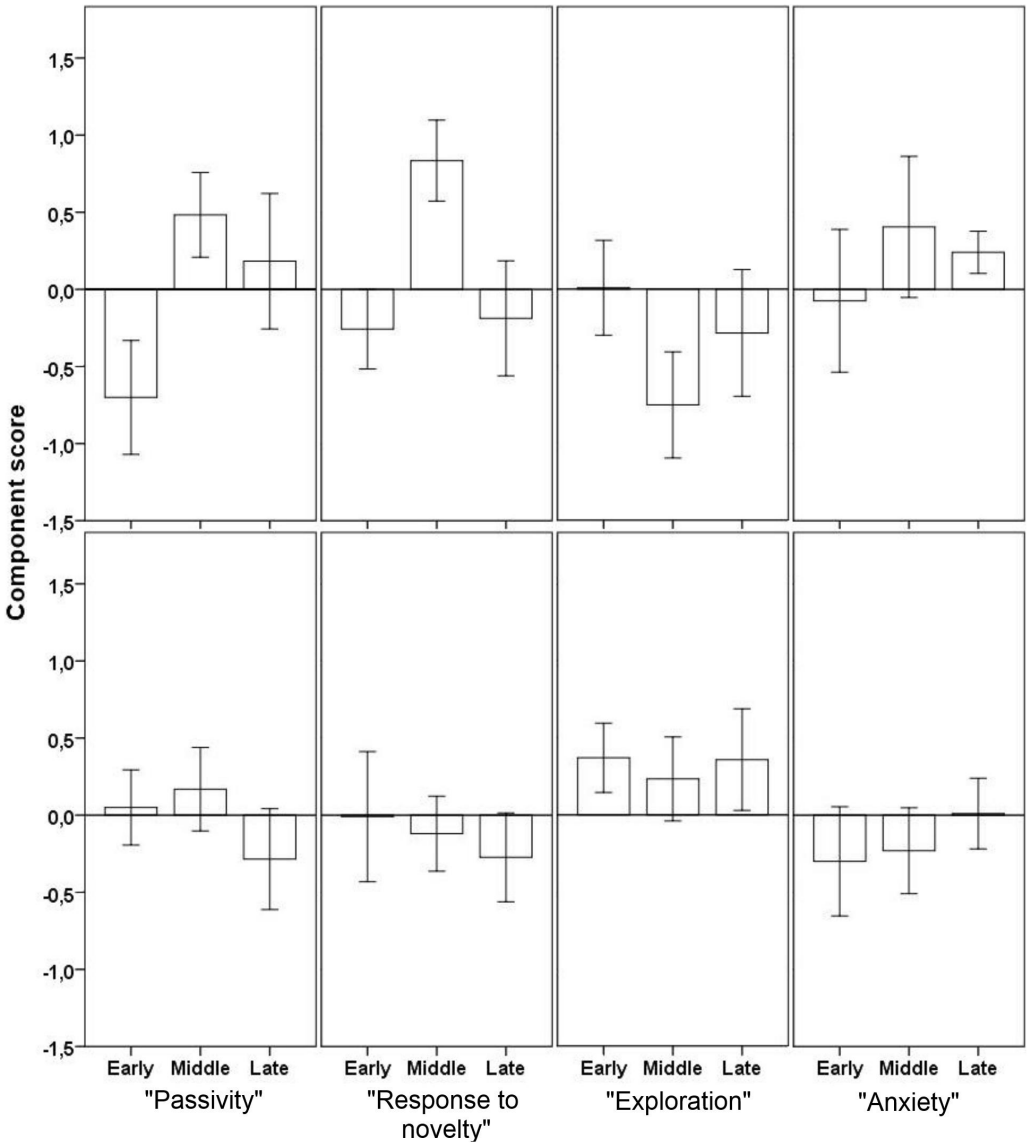
Component scores (Mean±S.E.) for each hatch group in the four components from the behavioral PCA. The top panel shows the results for the males, and the bottom panel shows the females.

Individual scores for each component were calculated, and average scores for males and females in the three hatch groups are shown in Figure 2. A significant difference between the sexes was found for “Exploration”, with the females scoring higher than the males (t(53) = -2.64, p = 0.01).

When analyzing data for both sexes together, none of the behavior components were significantly different between hatch groups (ANOVA, all p>0.05). However, in males only, there was a significant difference between the three hatch groups in the “Response to novelty” component (ANOVA, F_2,23_ = 4.04, p = 0.03). Post hoc tests revealed a tendency of a difference between early and middle hatchers (p = 0.05). The late hatchers were not different from either of the other groups.

There was also a tendency for a difference in the “Passivity” component (ANOVA, F_2,23_ = 2.69, p = 0.09), with the early hatchers having a numerically lower degree of passivity than middle hatchers.

In females, there were no significant differences between the hatch groups in any of the four components.

A significant correlation was found in males between the component “Passivity” and the weights on days 28 and later (r = 0.43 to 0.52, all p<0.05), and in females between the component “Exploration” and weights on days 35 and later (r = 0.39 to 0.43, all p<0.05).

### 3.4 Spatial learning

**Figure 3:**
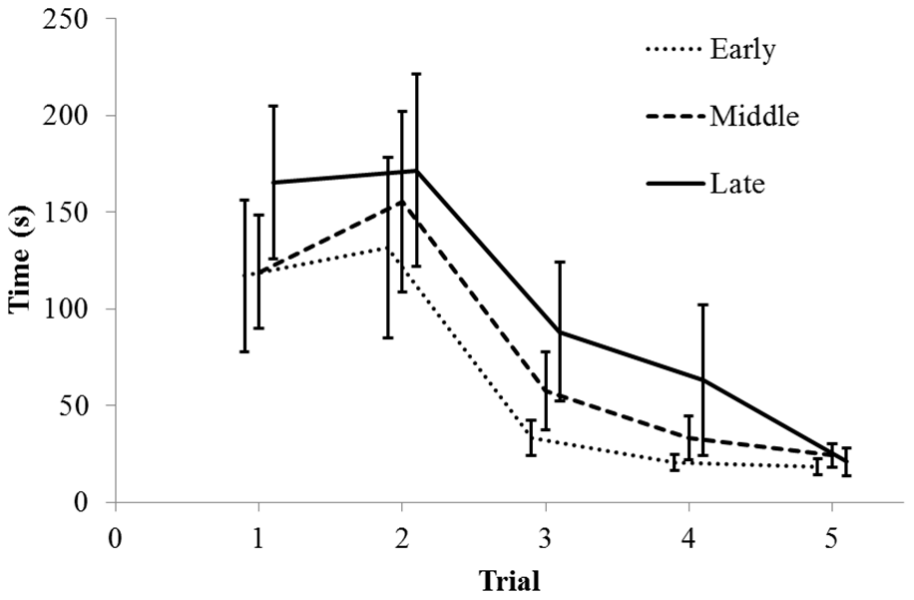
Average time (mean±S.E.) to reach the goal container in successive trials of the spatial learning test for each hatch group.

Spatial learning results were corrected for latency to start walking to remove confounding effects of fear. Figure 3 presents the latencies to reach the goal in each trial for each of the three hatch groups. All three hatch groups showed a significant decrease in time to reach goal over the five trials (Friedman test, all p<0.001). Following transformation (-1/x^0.5^) the time measures were normally distributed, allowing for repeated measures ANOVA. When all test trialswere included, there were no significant differences between hatch groups (F_2,33_ = 0.98, p = 0.39, N = 39). However, since animals were exposed to 5 trials in total, regardless of how many attempts it took to find the goal for the first time, several animals did not receive a score for 5^th^ successful trial, as well as for the 4^th^ successful trial. As a result, statistical analyses excluding animals with missing data, such as repeated measures ANOVA, would make better use of the data if the 5^th^ and the 4^th^ trials were removed from the analyses (N = 39 for all five trials, N = 47 for first four trials and N = 51 for the first three trials). For this reason, a repeated measures ANOVA was also performed with the first four trials only and the first three trials only. In the case of the first four trials, a tendency for a difference in the time to reach the goal over the first four trials (F_2,41_ = 2.77, p = 0.08, N = 47), with the early hatchers tending to solve the task more quickly than middle and late hatchers. When analyzing the males separately, there was also a strong tendency for early hatchers to finish earlier (F_2,18_ = 3.32, p = 0.06, N = 21), but this was not seen in females (F_2,23_ = 0.39, p = 0.68, N = 26).

For the first three trials only, a significant overall effect was found (F_2,45_ = 3.75, p = 0.03, N = 51), with post hoc tests showing that early hatchers finished earlier than late hatchers (p = 0.04). This was also seen in males (F_2,20_ = 3.60, p = 0.05, N = 23), with early male hatchers showing a tendency of finishing earlier than both middle (p = 0.09) and late (p = 0.07) male hatchers. In females only, there was no significant difference between the hatch groups (F_2,25_ = 0.60, p = 0.60, N = 28).

Finally, we also tested whether the distribution of animals reaching the different trials were different between the hatch groups and between sexes, using chi square analysis. There were no differences between hatch groups in whether or not the animals solved the task on the first attempt, if they solved the task at all, or the number of animals that reached any of the first four trials (χ^2^ = 0.73 to 2.22, df = 2, all p>0.1), and there was only a weak tendency for a difference in the number of animals that reached the 5^th^ trial (χ^2^ = 4.65, df = 2, p = 0.1). Neither were there differences in the number of males and females that solved the task or that reached any of the trials (χ^2^ = 0.22 to 2.31, df = 1, all p>0.1).

## Discussion

Time of hatching was related to post-hatch growth and behavior of chickens during the first 8 weeks of life, but males and females were affected differently. In females, body weight throughout the experimental period was negatively correlated with time of hatching, but no overall effect was seen on behavior. In males, on the other hand, a negative correlation between hatch time and body weight was only found when testing all hatched male chicks until day 10, whereas in the chicks kept for behavioral studies, only a tendency of such a correlation was observed, and only until day 14. On the other hand, several differences were found in behavior of males, particularly between early and middle hatchers. Early hatched males showed a higher response to novelty, and also showed a tendency of lower general passivity, as well as faster learning in a spatial learning task. This study is the first to show that chicks hatched at different times within the hatch window may display different behavioral phenotypes, and it is also in line with a number of previous studies demonstrating weight differences between early and late hatchers.

In the present study, we standardized as many of the pre-incubation factors as possible. Eggs were obtained from a single breeder flock from a commercial flock with minimal genetic variation, and the eggs were laid on the same day and not stored before incubation. All eggs were kept in the same incubator and hatcher, and treated in the same way. Despite this, there was a spread of hatch of 35.5 hours, similar to what has been observed in previous studies [10]. Unlike a number of other studies [10], [16], however, no difference was observed in the hatching times of males and females. Ichinoe [39] investigated hatching time in different chicken layer breeds and found that sex differences are considerably dependent on breed. To our knowledge, this has previously not been investigated in the Bovans breed, and it is possible that effects of both egg size and chick sex are not present in this breed.

No correlation was found between hatching time and egg weight or egg weight loss. In previous papers, results on the effect of egg weight are ambiguous, some finding a correlation [13]–[16], and others not [31]. On the other hand, hatch time was negatively correlated with hatch weight relative to egg weight. A previous study [10] reported lower residual yolk weights in late hatchers, whereas yolk free body mass was not affected. This could explain the decrease in relative hatch weight observed in this study.

### 4.1 Hatching time and post-hatch growth

Although no correlation between hatch time and hatch weight was observed, there was a negative correlation between hatch time and body weight starting on day 4. In females this correlation persisted throughout the 8 week long experiment, whereas no significant correlation or tendency of a correlation was found for males from day 14 and later.

Previous studies have also shown a similar pattern of decreased growth in late hatchers [10], [15], [18]. The reason for this lower post-hatch growth potential in late hatchers is currently unknown, but several studies have suggested a potential role of thyroid hormones [10], [15], [24]. Increased conversion of thyroxine (T_4_) to triiodothyronine (T_3_) levels appears to be a stimulus for hatching [9], and chicks with lower levels of T_3_ hatch later [10],[19],[24]. Lower thyroid hormone levels could in turn lead to lower post-hatch growth, due to interactions with growth hormone [40]. However, other studies have reached opposite results, showing higher thyroid levels in late hatchers [29]. Consequently, it is not yet known whether thyroid hormones constitute the link between hatching time and post-hatch growth.

Van de Ven [29] outlined a number of other possibilities that may contribute to differential post-hatch growth in early and late hatchers, such as differences in organ maturity at hatch, differences in yolk sac absorption and variations in blood glucose and lactate levels. However, the mechanisms behind these differences are unknown, and whether they do contribute to post-hatch growth differences is as of yet undetermined. It has also been observed that there are differences in the feeding behavior of early and late hatchers, with early hatchers feeding more [30], and this may be part of the reason why weight differences are observed. In conclusion, more research is needed to fully understand the link between hatching time and post hatch growth.

Previous studies showing a relation between hatching time and post hatch growth mostly do not mention sex differences [10], [15], [18]. However, Van de Ven [29] found that in broilers, early hatched males had larger growth potential than late hatching males, and no effect in females, contradictory to the current study. As a result, no conclusion can be made with regards to interaction between hatching time and sex when it comes to post-hatch growth.

### 4.2 Hatching time and behavior

When including both group and sex in the model, there were no significant effects of hatch group on the factor scores. However, since the sexes clearly differed in their responses to hatch time when looking at the individual behavior variables, we also analyzed the effects for the sexes separately. It was then found that early male hatchers displayed a higher response to novelty, as well as a tendency of lower passivity. Furthermore, early male hatchers showed a tendency for a slower spatial learning. All these effects may indicate that, in males, early hatchers are more fearful, which would affect their behavior much in the way observed. No effect of hatching time on behavior was found in females. This is the first study to investigate hatching time and behavior, apart from one investigation of feeding behavior [30], where an effect was found, supporting the notion that hatching time and behavior are related.

The findings indicate that hatching time may have both evolutionary and practical significance. In the wild, early hatching may be beneficial from the point of view of the incubating parent, but our results indicate that this comes at a potential cost of more fearful offspring. In practical settings, this may affect the way in which small chicks are able to cope with husbandry related stressors, such as transport and novel environments.

No mechanism explaining the relationship between hatching time and behavior is currently known, although hormonal variation is one likely possibility. In particular stress hormones and sex hormones are well known to exert large effects on the developing brain [41]. Similar to the thyroid hormones, corticosterone (CORT) has been implicated in the timing of hatching [42], and higher CORT levels might lead to earlier hatching [43]. Furthermore, CORT is one of the most well studied hormones in relation to effects of prenatal exposure on behavior [28], and it has been shown that prenatal treatment with CORT caused male chickens to be less aggressive and more prone to being pecked in comparison with controls, while no effect was seen in females [44]. Other studies have also shown larger effects on males than females [27], [28], and thus, prenatal CORT levels may affect both hatching time and behavior, the latter in a sex dependent manner.

However, not all studies have found differences in CORT levels with different hatching times [10], and further studies investigating the relationship between CORT, hatching time and behavior are needed, as well as investigations of other underlying factors that may explain the link between hatching time and behavior.

It should be noted that the percentage of variance explained for each variable in the behavioral PCA is somewhat low. However, this is not uncommon with behavioral tests [37], [45]–[47]. In fact, the four PC:s extracted explained 62.9% of the total variance, which is considerably higher than observed in several comparative studies on chicken behavior (for example, [37], [45]).

### 4.3 Implications of the findings

We have demonstrated several sex dependent phenotypic effects of hatch time variation on the phenotypes of young chickens, and this may have both practical and theoretical implications. For example, numerous studies have investigated consequences of asynchronous hatching in different species, where the mother starts incubating the eggs before the entire clutch has been laid, causing some eggs to hatch earlier than others. In this situation, large phenotypic differences have been found between early and late hatchers. Among the affected traits are differences in growth [48], survival [49], HPA-axis activity [50], sexual attractiveness [51], and adult behavior [52]. It is possible that some of the long-term effects observed in asynchronous species would be apparent also in artificially hatched batches of chicks, which is usually considered to be a synchronous hatcher, since there obviously is a considerable variation in incubation length also in this species. Indeed, effects were seen on both growth and behavior, and previous studies also indicate differences in mortality [53]. Whether or not these phenotypic similarities are due to similar mechanisms in asynchronous hatching and artificial incubation remains to be seen.

As discussed in previous sections, we observed sex-dependent effects. Differences in post-hatch growth were mainly seen in females, whereas behavior was mostly affected in males. As relatively little work has been done in this area, it is difficult to draw conclusions on this, and more investigations are needed to determine to which extent the sexes are affected differently. If it is indeed a widespread effect, this may have implications for research on for example maternal programming, where the mother may want to program the offspring differently depending on their sex [27].

For the effects on both post-hatch growth and behavior, hormones are suggested as potential mechanisms underlying the observed differences in hatching time and other traits. Whether these hormones are a result of the individual's own hormone production, or if they may be a result of maternal egg hormone deposition is not known. However, experiments have shown potent effects of injecting eggs with various hormones, including sex hormones and stress hormones [27], [28]. Furthermore, implantation of hormones in egg-laying hens have shown that this affects hormone content in the egg. It is therefore possible that mothers can affect traits such as hatching time, growth and behavior through hormone deposition into the egg [27], [28]. But it is also possible that the embryo can be affected by environmental stimuli, causing it to alter its own hormone production and thereby affecting future traits [28], including hatching time, growth and behavior. If mechanisms such as these are in play, more studies will be needed to elucidate them.

In the present study, the chickens were only kept until the age of 8 weeks. Therefore we cannot draw conclusions concerning the effects of differences in hatching time on adult chickens. Previous studies have focused on chicks during the first 7 days of life [15], [18], or up until slaughter age of broilers [10]. Certainly, the effects may be diminished with age. However, numerous studies have shown long lasting effects of other pre- and perinatal experiences, such as early stress [54] or exposure to maternal hormones [27]. Whether or not long-lasting effects are seen in relation to different hatching times remain to be investigated.

Investigations of the effect of differences in hatching times may contribute to increased understanding of how individuals within a batch differ from each other. This is an important aspect for commercial hatching industries, where the goal is to achieve a homogenous and well-performing batch. Furthermore, studies have demonstrated an interaction between the time of hatching and the time until the chick has access to food and water [15], [42]. In commercial settings, hatched chicks are removed from the incubator only at a point when most eggs have already hatched, leading to differences in the time from hatching to feeding. Early hatchers thus spend longer time in the sub-optimal hatcher environment, and this also affects them differently than late hatchers when it comes to post hatch growth development [15]. Whether or not this also interacts with shaping of behavior is not yet known, but it may also be another factor decreasing homogeneity of a batch. Finally, the results of this and previous studies do not agree with the notion that incubation length in chickens is a trade off with developmental maturity at hatch, as has been suggested. Instead, late hatchers appear to have lower growth potential and have been suggested to score lower in chick quality systems than early hatchers [15]. Length of incubation is probably governed by underlying, partly unknown factors that affect both this trait and post-hatch phenotypes, and further studies are needed to fully understand these mechanisms.
